# Effect of an Alltech soil health product on entomopathogenic nematodes, root-knot nematodes and on the growth of tomato plants in the greenhouse

**DOI:** 10.21307/jofnem-2020-014

**Published:** 2020-03-19

**Authors:** Anusha Pulavarty, Karina Horgan, Thomais Kakouli-Duarte

**Affiliations:** 1 Molecular Ecology and Nematode Research Group, enviroCORE, Department of Science and Health, Institute of Technology Carlow, Kilkenny Road, Carlow, Ireland; 2 Alltech Bioscience Centre, Dunboyne, County Meath, Ireland

**Keywords:** Entomopathogenic nematodes, Root-knot nematodes, Tomatoes, Bioassay

## Abstract

An organic product that consists of proprietary blend of fermentation and plant extracts with micronutrients (ACS 5075, Alltech^®^, Inc., Nicholasville, KY USA) was evaluated against four strains of entomopathogenic nematodes (EPN): *Steinernema feltiae* (SB12(1), a wild enviroCORE strain and a commercial form e-NEMA), *Steinernema carpocapsae* (e-NEMA), and *Heterorhabditis bacteriophora.* The effects on egg hatching and survival of root-knot nematodes (RKN) were also examined. The sensitivity to the product was tested by estimating mortality and survival of EPN infective juveniles (IJ) after 24-hr treatment with four different concentrations of product (4, 7, 8, and 10%) compared with the control in a 96-well plate. There was no significant difference in survival of IJ with 4% product compared to the control. A twofold reduction in survival was observed when the EPN were exposed to the product at 7%. A 10.5% RKN egg hatching was observed when RKN were exposed to 3% ACS 5075 concentration compared to 100% hatching in the untreated. A significant (*p* < 0.05) reduction in juvenile survival was observed at 0.5% treatment, however, it dropped to 0 when 1% and above was used for *M. javanica*. Juveniles did not survive with all treatment concentrations in the case of *M. incognita.* The improvement in tomato plant growth and development were also observed when plants were treated with 1 and 3% ACS 5075. The product shows potential as a sustainable soil health alternative causing no harm to beneficial nematodes (EPN) at concentrations below 4%, while is promising against plant parasitic nematodes (PPN) and toward plant growth promotion.

In the context of food security for an ever increasing global population, nematodes pose serious threat to agricultural crops worldwide (Miamoto et al., 2017). There are nearly 4,100 species of plant parasitic nematodes (PPN) reported to date that are currently a serious constraint for global food security (Jaouannet et al., 2013) causing yield loss of about 30% in susceptible crop varieties annually (tomatoes, eggplant, and melons) (Padgham et al., 2004; Collange et al., 2011). Among these root-knot nematodes (RKN) are widely spread and highly damaging (Zakaria et al., 2013). The four predominant species are *Meloidogyne arenaria*, *M. incognita*, *M. javanica*, and *Meloidogyne hapla* (Jones et al., 2013). De Waele and Elsen (2007) have reported the difficulty in controlling the damage caused by *Meloidogyne* species due to their short life cycle and broad host range.

Biological control is a highly preferred, effective, non-polluting, and environmentally safe approach that should be considered while adopting any sustainable pest management approach (Saleh et al., 2017). Very often, in an attempt to control PPN, the beneficial entomopathogenic nematodes (EPN) are harmed by bionematicides or organic amendments (Bednarek and Gaugler, 1997; Somasekhar et al., 2002). Entomopathogenic nematodes are widely used by farmers and growers commercially for biological control of insect pests (Somasekhar et al., 2002). Species within the EPN genera *Steinernema* and *Heterorhabditis* (Rhabditida) are widely utilized as biocontrol agents in crop protection (Zakaria et al., 2013).

Therefore, simply identifying harmful nematodes and applying a nematicide is not a permanent or long-lasting solution for nematode management, in the context of conserving non-target and beneficial nematodes, while in many cases safe potential nematicides are not available. Conventional chemical nematicides are expensive, carcinogenic, and toxic to humans, animals, and the environment. Moreover, unfavorable climatic conditions can make the applied nematicide ineffective against nematodes.

Multiple management strategies such as use of organic amendments, soil solarization, and nematicide application were also adopted to protect tomato plants against the *Meloidogyne* species (Terefe et al., 2009). Among these strategies, nematicides were successful up to certain extent, but they were very expensive and led to soil pollution problems. Youssef and Eissa (2014) have studied the role of biofertilizers in the management of PPN. Limitation with the use of biofertilizers would be environmental conditions like soil temperature, pH, and moisture, as the microbes associated with biofertilizers may not be effective until and unless these factors are favorable.

Organic amendments are promising solutions to control PPN growth and may influence other beneficial soil organisms (Mcsorley, 1998; Oka, 2010; Lord et al., 2011). In some studies, the use of organic amendments has positively suppressed the levels of nematodes in the soil, and has resulted in increased levels of nematode trapping fungi or other potential predators or parasites of PPN in the same soil (Akhtar and Malik, 2000; Wang et al., 2001; Oka, 2010). Bednarek and Gaugler (1997), however, have reported the reduced efficacy of EPN while applying organic amendments to soil. Therefore, thorough research is essential to study the long- and short-term effects of any amendments to both groups of nematodes prior to application.

Global awareness on sustainable production has increased demand for bionematicides. The leading multinationals in the market are Bayer crop science, BASF, Agri life, Dow AgroSciences, Valent Biosciences, etc. Alltech Crop Science is one such multinational company that aims to produce natural-based solutions to the agricultural problems faced by growers worldwide. South and east Asia, North America, and Europe have been showing significant value share in the production and application of organic nematicides due to ascending agricultural activity and eradication of chemical fertilizers (Bionematicides market report – Global industry analysis 2014-2018).

Products obtained from natural fermentation process such as abamectin (Khalil, 2013) and those from solid state fermentation of *Verticillium chlamydosporium*, *Hirsutella rhossiliensis*, and *Paecilomyces lilacinus* (González Chávez and Ferrera Cerrato, 2001) have also been reported to be effective against PPN. These products have been produced in large scale and have been commercialized by German manufacturer Prophyta for application in several countries. Natural plant extracts from tobacco, cloves, garlic, neem, soyabean, capsicum, betelvine, castor, and lemon grass have been tested against PPN and were found to possess larvicidal and ovicidal properties (Korayem et al., 1993; Adegbite and Adesiyan, 2006; Wiratno et al., 2009; Kepenekçi et al., 2016), but their effect on beneficial soil microbes was not reported. To the best of our knowledge, there have been no studies conducted using bionematicides/organic amendments that could be effective against PPN while displaying no negative impact on beneficial EPN. This research is therefore the first of its kind in this context.

This study is designed to examine the effects of a proprietary soil health product blend of fermentation and plant extracts with micronutrients (ACS 5075, Alltech^®^, Inc., Nicholasville, KY USA), on the egg hatching and survival of the root-knot nematodes *M. javanica* and *M. incognita*, while studying its compatibility with EPN, and monitoring its effects on the growth of tomato seedlings (Fig. 3). This approach of considering the compatibility of product with beneficial nematodes makes the study unique and could serve as a viable solution for sustainable PPN management.

## Materials and methods

### Culturing and maintenance of EPN

Four EPN strains of the family *Steinernematidae* and *Heterorhabditidae* were cultured, maintained, and stored at 9°C in the enviroCORE laboratory facilities, Institute of Technology Carlow, Ireland. These were: an Irish isolate *Steinernema feltiae* [strain 12(1); Boyle, 2007], *Steinernema feltiae* (e-nema), *Steinernema carpocapsae* (e-nema), and *Heterorhabditis bacteriophora* (Andermatt Biocontrol UK).

Entomopathogenic nematodes were reared in *Galleria mellonella* (Lepidotera: Pyralidae), sourced commercially from Live Foods Direct (Sheffield, UK). Standard size petri dishes (100 × 15 mm) were inverted and the lids were lined with two sheets of Whatman filter paper. Five *G. mellonella* were placed onto the filter paper, the base was placed on top. Approximately 1 to 1.5 ml of a dense IJ suspension was applied to the filter paper until it was moist. The dish was lightly sealed with parafilm to prevent the filter paper drying out. Plates were then incubated in the dark, at 21°C for three to seven days until mortality occurred.

Once insect mortality had occurred, nematode IJ were recovered using White traps (White, 1927). Freshly hatched juveniles were then stored at 9°C until required and were used for experimentation no more than two weeks post emergence.

### EPN bioassay to determine lethal and sub-lethal concentrations of ACS 5075, Alltech^®^

All the treatment studies were conducted in 96-well plates. Freshly cultured EPN, not older than two weeks post emergence, were used for the study. EPN were kept in room temperature for 30 min before conducting the experiments to check their viability. The four EPN strains were concentrated by transferring the cultures into sterile and clean falcon tubes. The falcon tubes were kept upright, to allow the IJ to settle down at the bottom of the tube. Slowly, the supernatant was decanted to obtain approximately 10  IJ/100 μl. In all, 100 μl of water containing approximately 10 juveniles and 100 μl of the various product (ACS 5075 obtained from Alltech^®^ Inc) dilutions were added in each well to obtain the required concentration. In case of 100% treatment concentration, the product was directly added without any dilutions and the juveniles were picked up individually by observing under the stereoscope using micropipettes. The concentrations of product tested were 0, 10, 20, 40, 60, 80, and 100% in a total volume of 200 μl in each well of the 96-well plate. The Experiments were conducted to determine the lethal concentration of the product for each EPN strain. Furthermore, concentrations within the range of 0, 2, 4, 6, 8, and 10% were used to determine the sub-lethal concentration (LC_50_) of the product for each EPN strain. All the experiments were conducted with 10 replicates for each treatment concentration and the untreated (control), which received water. Each set of experiments was carried out three times.

After 24-hr incubation at 20 to 22°C, survival and mortality were calculated by counting the motile and immotile IJ using an Olympus Stereo Microscope (SZX7). The mortality of immotile juveniles was ensured by gently touching them with a needle. Another set of plates with the same concentration range was analyzed after 48-hr incubation. It was observed that the mortality level was similar to that of 24-hr incubation. Therefore, data recorded after 24-hr incubation were considered for statistical analysis. All these bioassays were repeated two more times.

### Sourcing and establishment of root-knot nematode cultures

Tomato roots infected with *M. javanica* and *M. incognita* were a kind offer from Dr E. A. Tzortzakakis, Hellenic Agricultural Organization – DEMETER, Greece. Three- to four-week-old tomato seedlings were freshly infected with five to seven egg masses/plant around the root, for rearing and increasing the nematode population for further studies. All the plants were maintained in a plant growth room at 32 ± 2°C. Organic pesticide was sprayed on the leaves and stem to avoid white flies, spider mites, and insect infection. Tomato roots were assessed for nematode galling damage approximately after a period of 8 to 12 weeks. Egg masses were excised from the roots with the help of sterile scalpel and forceps and were stored at 9°C to cease hatching before application of the actual treatment. All PPN work, in culturing and in experimentation, was carried out under strict quarantine and containment conditions.

### Preparation of nematode inoculum: egg hatching from egg masses

Experiments were conducted to collect nematode inoculum by hatching juveniles from the egg masses collected from infected tomato roots. The protocol was a slightly modified version of the one followed by Coyne and Ross (2014). The egg masses were removed carefully using a scalpel and fine tweezers. They were placed on moist filter paper, which was in turn placed in a plastic container with 20 ml of distilled water. The container was incubated in dark at 20°C for 48 hr for juveniles to emerge out into the water.

### RKN egg hatching and juvenile mortality assay

Five concentrations of product were prepared in 5 ml water, i.e., 0% (control), 0.5%, 1%, 2%, and 3% and placed in 35 mm sterile petri dishes. Five similarly sized egg masses were randomly picked up and placed in each petri dish containing respective treatment concentrations. Egg masses placed in sterile water were considered the control. These petri dishes were incubated in dark at room temperature and checked for hatching at regular intervals of 24 hr for three days. After three days of treatment, the suspensions in each treatment were thoroughly mixed and placed in a counting dish each, to record the number of juveniles hatched using Olympus Stereo Microscope (SZX7).

Freshly hatched RKN juveniles (J2) obtained three days after incubating egg masses at room temperature were used to study the effect of product on juvenile mortality. The study was conducted in a 96-well plate. In total, 100 μl of inoculum containing approximately 10 juveniles and 100 μl of the product dilutions were added to each well to obtain the required concentrations, i.e., 0.5, 1, 2, and 3%, respectively. In total, 100 μl inoculum containing 10 juveniles with only water was used as control and 10 replicates were run for each concentration.

After 24-hr incubation at 20 to 22°C, in the dark, mortality was calculated by counting the motile and immotile juveniles using Olympus Stereo Microscope (SZX7) and expressed as a percent relative to the control. The mortality of immotile juveniles was ensured by transferring them into separate petri dishes containing water without product and were observed after 24 hr. Mortality was further confirmed after that. Another set of plates with the same concentration range was analyzed after 48-hr incubation, and the mortality level was similar to that of 24-hr incubation. Therefore, data recorded after 24-hr incubation were considered for statistical analysis. All these experiments were repeated two more times.

### Treatment of tomato plants in soil

Tomato (*Solanum lycopersicum*) seeds were germinated under environmentally controlled glasshouse conditions at 32 ± 2°C, 70 ± 10% relative humidity (RH) and natural day/night cycle. The seeds were first grown on garden soil with the following characteristics: pH-7.6 ± 0.02, electrical conductivity- 540 μs/cm, clay (%) 8 ± 0.02, silt (%) 19 ± 0.5, sand (%) 73 ± 3.9, Na 12.58 (mg Kg^−1^), P 2.8 (mg Kg^−1^), K 30.59 (mg Kg^−1^). The seedlings attained a height of around 6 to 10 cm before taken for treatments. They were carefully removed from the soil bed and were transplanted into the individual soil pots. Seedlings were grown in plastic pots with 1 kg soil mixture with a composition of soil: compost ratio being 1:1 ratio.

Treatment ACS 5075 was in range of 0, 1, 3, 5, and 7% prepared in water. Product dilutions were prepared in a total volume of 100 ml and were poured into the plastic pot saucers. These pot saucers were placed beneath the tomato plants growing pots. Each pot had several perforations in the bottom allowing the roots of the tomato seedlings to take up the solutions from the saucers that were placed under the pots. Product treatment was applied only once at the start of the experiment. Tomato plants were regularly irrigated with equal volumes of water. Treatments including control pots were conducted in triplicates, after the treatment duration of 1 month, seedlings were harvested to measure the growth parameters.

### Measurement of growth parameters and determination of chlorophyll content

Three tomato seedlings of same age and variety were randomly selected for treatment study. The treatment was conducted as described above. The treated and control seedlings were irrigated at the same time with equal volumes of water and were monitored regularly.

The effects of product on growth of tomato seedlings were studied in terms of percent increase in shoot length (SH) and number of leaves (NL) from the day of treatment (0th day) up to 30 days.

Shoot height (SH) (cm) and number of leaves (NL) of plants were recorded. SH (cm) was measured using meter rule and NL by visual counting.

The chlorophyll content (Total Chlorophyll (TC), Chlorophyll_a_ (Chl_a_), and Chlorophyll_b_ (Chl_b_)) was determined to study the effect of treatment on tomato plants (Pulavarty and Sarangi, 2018). Approximately 250 mg of leaf samples were weighed and macerated with 10 ml of 80% acetone using a mortar and pestle. The contents were centrifuged at 3,000 rpm for 10 min. The supernatant was collected, and its volume was made up to 25 ml using 80% acetone. Light absorbance was measured at 480, 510, 645, 652, and 663 nm by UV–visible spectrophotometer (UV-1800, Shimadzu UV spectrophotometer). Chl_a_ was calculated using the formula: (12.7 × OD at 663) − (2.69 × OD at 645) × v/1,000 × weight of the sample taken in gm. Chl_b_ content was calculated by: (22.9 × OD at 645) − (4.68 × OD at 663) × v/1,000 × weight of the sample taken in gm. TC content was calculated by: {(ABS_652_ × 1,000)/34.5) × (Final volume of supernatant/1,000 × weight of leaf sample taken in gm)} = mg chlorophyll g^−1^ FW (Pulavarty and Sarangi, 2018).

### Statistical analysis

All the experiments were statistically designed and analyzed. The experiments were arranged in a completely randomized factorial design (CRD). Dose-response bioassays were performed in 96-well plates with 10 replicates for each treatment including control (only water with no product). These bioassays were repeated two more times. Mortality data were recorded manually for each treatment and were subjected to probit regression analysis using IBM SPSS version 23 (Tzortzakakis, 2017). The software transforms the sigmoid dose-response curve into straight line by converting mortality units into probits and analyzing them against the log of concentration. The regression slope provides the sub-lethal concentration (LC_50_) at which 50% mortality was monitored. The mean values obtained from the plant experiments were separated based on least significant differences (LSD) obtained from analysis of variance (ANOVA) using IBM SPSS version 23. The plant experiments were also repeated two more times.

## Results

### EPN bioassay to determine lethal and sub-lethal concentrations

There were no statistically significant different results recorded among the experiments carried out over repeated times in any type of bioassay and the results were reproducible.

#### Effect on *S. feltiae* (SB 12(1)) and *S. feltiae* (e-nema)

**Figure 1:**
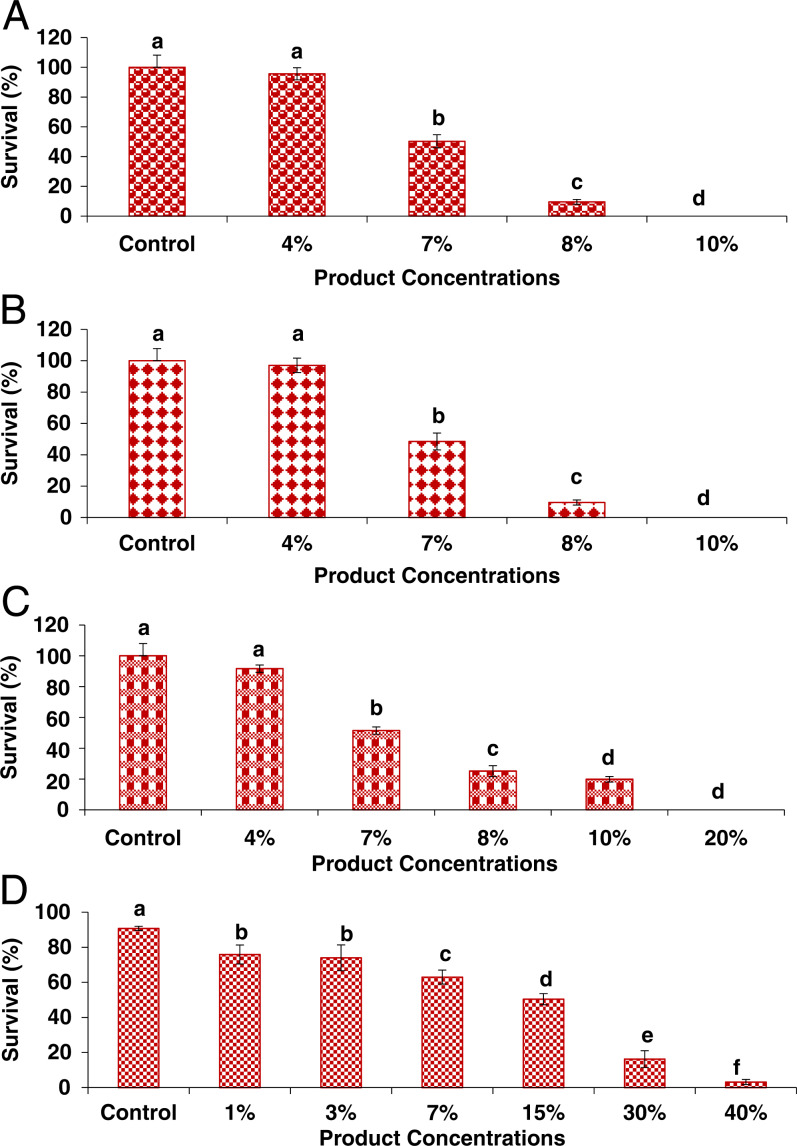
The impact of varying concentration of Alltech ACS 5075 on viability of EPN species in vitro (A) *S. feltiae* (SB 12(1)), (B) *S. feltiae* (e-nema), (C) *S. carpocapsae*, (D) *H. bacteriophora* survival. Data were analyzed using one-way ANOVA with significant difference compared to the control (*n* = 10). Values represented by similar letters are not significantly different from each other (*p* ≤ 0.05).

ACS 5075 product had a significant and strong effect on *S. feltiae* (SB 12(1)) and *S. feltiae* (e-nema). In total, 100% mortality was noted at concentrations above 10%. In case of *S. feltiae* (SB 12(1)), survival was 100, 95.6, 50.2, 9.5 and 0%, for the control, 4, 7, 8, and 10% product concentrations, respectively (Fig. 1A), whereas survival was 100, 97.07, 48.5, 9.5, and 0% for control, 4, 7, 8, and 10% product concentrations, respectively, for *S. feltiae* (e-nema) (Fig. 1B). There was no significant difference in survival percentage of IJ for the 4% concentration when compared to that of control conditions. However, survival decreased with the increase in product concentrations; 2- to 2.06-folds and 10-fold decreases in survival at 7 and 8% ACS 5075, respectively, when compared to control were observed. At 10%, ACS 5075 no *S. feltiae* survived. The use of probit analysis determined 6.71 and 6.73% as LC_50_ concentration for *S. feltiae* (SB 12(1) and *S. feltiae* (e-nema), respectively.

#### Effect on *S. carpocapsae* (e-nema)

A significant (*p*-value < 0.05) effect on *S. carpocapsae* (e-nema) was observed with 100% mortality at the 20% concentration of ACS 5075. Survival was 100, 91.6, 51.4, 25.1, 19.8, and 0%, for control and 4, 7, 8, 10, and 20% of product, respectively (Fig. 1C). There was no significant difference in survival of IJ for the 4% product when compared to that of control conditions. However, survival decreased with the increasing product concentrations. It was observed that there was 1.94-fold, 3.98-fold, and 5.03-fold decreases in survival at the 7, 8, and 10% product, respectively, when compared to the control. At the 20% concentration, no *S. carpocapsae* (e-nema) survived. The LC_50_ concentration of ACS 5075 was 6.79% for *S. carpocapsae* (e-nema).

#### Effect on *H. bacteriophora*

A significant (*p*-value < 0.05) effect on *H. bacteriophora* was observed, 100% mortality was observed when *H. bacteriophora* was exposed to 40% ACS 5075. In all, 90.8, 75.9, 73.9, 63, 50.4, 16.2, and 3.1% of *H. bacteriophora* juveniles survived, when exposed to control, and 1, 3, 7, 15, 30, and 40% of product, respectively (Fig. 1D). Survival was found to decrease with increasing products concentrations, and 1.2-fold, 1.2-fold, 1.4-fold, 1.8-fold, 5.6-fold, and 29.3-fold reductions in survival were recorded when juveniles were exposed to 1, 3, 7, 15, 30, and 40% concentrations, respectively, compared to control. In all, 40% of the product was considered as lethal concentration, whereas 15% was considered as LC_50_ for *H. bacteriophora* using probit analysis.

### Effect of product on growth parameters and chlorophyll content of tomato seedlings

**Figure 2:**
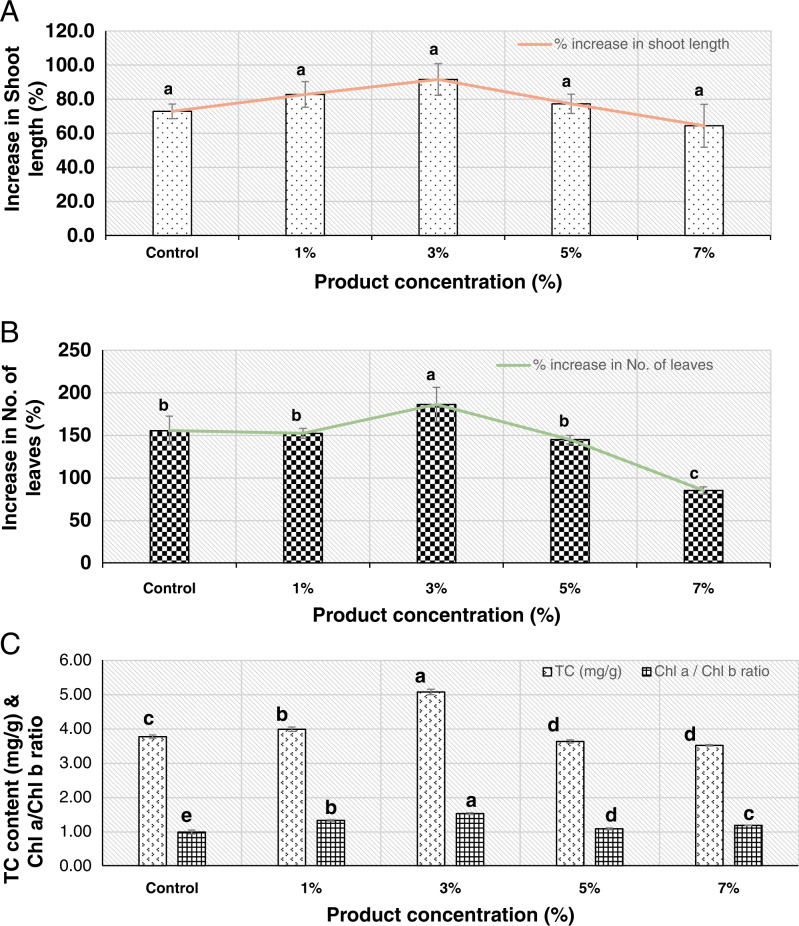
Effect of ACS 5075 on: (A) percentage increase in shoot length of tomato plants, (B) percentage increase in number of leaves, and (C) chlorophyll content. Data were presented as mean ± SEM (*n* = 3). Values represented by similar letters are not significantly different from each other (*p* ≤ 0.05).

The seedlings treated with 3% product had the highest increase in shoot length (91.6%) followed by 1% (82.8%), 5% (77.3%), control (72.9%), and 7% (64.4%) treatment concentrations, respectively, however these values were not significantly different from control (Fig. 2A).

A similar but significant effect (*p*-value = 0.00) was observed in terms of number of leaves (Fig. 2B). The seedlings treated with 3% product had the highest increase in number of leaves (186.1%) followed by the control (155.4%), 1% (152.2%), 5% (144.8%), and 7% (85%), respectively, whereas 1 and 5% treated seedlings had no significant effects compared to control seedlings. At 7% ACS 5075, there was a slight decline (1.8 fold) in plant growth in terms of number of leaves when compared to those in the control plants.

Total chlorophyll (TC) content and the Chl_a_/Chl_b_ ratio were significantly affected (*p*-value = 0.000) in treated plants compared to ratios in the untreated (Fig. 2C). Tomato plants treated with 3% product had the highest TC (5.07 mg g^−1^) and Chl_a_/Chl_b_ ratio (1.52). In the cases of the untreated and the 1, 5, and 7% treated plants, TC and Chl_a_/Chl_b_ ratios were 3.77, 3.98, 3.63, and 3.51 mg g^−1^ and 0.98, 1.32, 1.08, 1.18, respectively. There was a 1.35- and 1.06-fold increase in TC content in 3 and 1% treated plants when compared to the TC content in the control plants (Fig. 2C). However, TC content and Chl_a_/Chl_b_ ratio (Fig. 2C) in 5 and 7% treated plants were slightly reduced when compared to those in the untreated plants.

### *M. javanica* and *M. incognita* egg hatching and juvenile mortality assay

A significant (*p*-value = 0.02) effect on both egg hatching and juvenile mortality in both the species of RKN was noted. In the case of *M. javanica*, 9.6-fold and 31.6-fold reductions in egg hatching were observed with 0.5 and 1% ACS 5075, respectively, treated egg masses when compared to that of control after 72 hr of treatment. At concentrations above 1%, the hatching process completely ceased. Similarly, there was 100% survival in the untreated, but this declined to 10% in the 0.5% ACS 5075 treated juveniles and 0% in the case of all other concentrations above 0.5% after 24 hr of treatment (Table 1).

**Table 1.** RKN egg hatching and juvenile survival following exposure to ACS 5075.

	*M. javanica*		*M. incognita*	
Product concentration (%)	No. of juveniles hatched (J2 juveniles/ml)	Survival (%)	No. of juveniles hatched (J2 juveniles/ml)	Survival (%)
Control	220 ± 10 a	100 ± 0.8 a	200 ± 12 a	100 ± 9 a
0.5	23 ± 5 b	10 ± 0.5 b	0 ± 0 b	0 ± 0 b
1	5 ± 0.5 c	0 ± 0 c	0 ± 0 b	0 ± 0 b
2	0 ± 0 d	0 ± 0 c	0 ± 0 b	0 ± 0 b
3	0 ± 0 d	0 ± 0 c	0 ± 0 b	0 ± 0 b

Notes: Values represented by similar letters are not significantly different from each other (*p*⩽0.05). Data were presented as mean±SEM (*n*=10).

In the case of *M. incognita*, the product had a strong and significant (*p*-value = 0.012) effect on both egg hatching as well as juvenile survival (Table 1). At all the concentrations above 0.5%, no egg hatching or juvenile survival was recorded compared to control after 24 hr of treatment.

## Discussion

In this work we have tested the efficacy of an Alltech® crop science soil health product against the RKN *M. javanica*, *M. incognita* and have also studied its effect on EPN and the growth of tomato seedlings. From this study, we determined the lethal and sub-lethal concentrations (LC_50_) of this product for several EPN strains, and tested the effectiveness of the product against PPN, at concentrations below that LC_50_ limit at which EPN are unaffected.

The initial studies conducted to find the lethal and sub-lethal concentrations (LC_50_) of a proprietary blend of fermentation and plant extracts with micronutrients against all four strains of EPN have clearly shown 6.7, 6.79, and 15% of the product as the LC_50_ concentration for *S. feltiae*, *S. carpocapsae*, and *H. bacteriophora*, respectively. At concentrations 4% and below, there was no or very negligible IJ mortality and the survival percentage was similar to that of IJ under untreated conditions. From this point, product concentrations of 4% and below were considered safe for EPN. The presence of EPN in soil not only manages insect pests but also induces systemic resistance in plants, by which a plant can inherently attain resistance against PPN infestation (Jagdale et al., 2009). Toxins and antibiotics produced by the EPN symbiotic bacteria are reported to have bactericidal, fungicidal, and nematicidal properties that could be effective against PPN (Isaacson and Webster, 2002). The presence of EPN in soil has contributed in reduction of PPN infection either by producing toxins, or by stimulating growth of PPN antagonists and predators (Ishibashi and Choi, 1991), or by releasing certain secondary metabolites that could inhibit the egg hatching process in *Meloidogyne* species (Jagdale et al., 2009).

Based on the concentration of the product that is compatible to EPN, all further experiments were designed at concentrations below 4%. The effect of this novel product on the growth of tomato plants, at four different concentrations, compared to growth in untreated plants, was assessed. The effect of the product, studied in terms of morphological growth parameters, clearly showed a positive contribution at 1 and 3% treatment concentrations, when compared to growth of untreated control plants. The increase in number of leaves of the treated seedlings corelated with the increase in their net chlorophyll content. The product had a significant effect on the chlorophyll content of the treated plants up to the 3% treatment. The presence of micronutrients is important to aid plant chlorophyll production (Thalooth, 2006). Plants treated with 3% product had the highest chlorophyll content and Chl_a_/Chl_b_ ratio. This is very beneficial for the plant as increase in chlorophyll content could contribute to increase in net photosynthetic rate (*P*_*n*_) and in turn could enhance the overall plant growth (Pulavarty et al., 2016).

The PPN undergo complex developmental stages for about 4 to 8 weeks that include an egg and four juvenile stages (J1-J4) before emerging as male or female adults (Khan et al., 2006). The second-stage juveniles emerge from eggs and travel in water films beneath soil and root surfaces to find and infect roots or foliar tissues. A product can be considered to have great potential in control of PPN if it can interfere with the life cycle and cease the process of egg hatching (Zakaria et al., 2013). In the current investigation, we tried to explore the efficacy of the proprietary blend of fermentation and plant extracts with micronutrients against the PPN egg hatching process. The product was found very effective by inhibiting the hatching process, as the egg masses treated with concentrations 0.5 and 1% had 9.6-fold and 31.6-fold reduction in egg hatching compared to the untreated (water with no product) egg masses. By preventing the process of egg hatching, approximately 500 to 1,000 juveniles could be potentially obstructed from further infecting the same or nearby roots and starting a fresh life cycle (Terefe et al., 2009). The efficacy of the product in controlling the egg hatching process could be due to its organic carbon content (Miamoto et al., 2017), however, the exact reason is yet to be explored. Although approximately 23 ± 5 and 5 ± 0.5 juveniles of *M. javanica* hatched in 0.5 and 1% treatment solutions, respectively, they were found to be immotile or dead. In addition, the hatching process was completely ceased at all the treatment concentrations in the case of *M. incognita*, indicating the promising potential of the product for the management of this PPN species.

**Figure 3:**
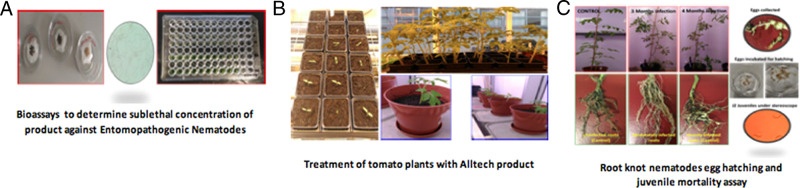
(A-C) Outline of Experiments.

The study on juvenile survival, as affected by the product, noted a 10-fold reduction in juvenile motility at the 0.5% treatment, and this was reduced to zero in concentrations 1% and above (Table 1). Juveniles of *M. incognita* showed a very low motility and survival level. The combination of fermentation products and plant extracts may be potentially having these effects on juveniles, as a similar effect was observed while evaluating BioNem, another biological product, against *M. incognita* juveniles (Terefe et al., 2009).

Sustainable PPN management is a huge challenge to growers worldwide due to their broad host range and vast crop losses (Vieira dos Santos et al., 2012). The major drawback of using bionematicides is that they affect non-target, beneficial organisms that could serve as biological control agents in sustainable agriculture (Korthals et al., 2014). A study in Florida illustrated the impact of applying organic amendments and reported the difficulty in maintaining one beneficial group of organisms in a particular soil (McSorley, 2011). EPN constitutes such economically important biological control organisms. These are highly beneficial nematodes that have been drastically affected in the process of controlling PPN (Bednarek and Gaugler, 1997). Protecting or safeguarding these insect pest biological control agents is a prime requirement for maintaining a balanced ecosystem. There are no studies conducted to date, that the authors are aware of, with any product which is effective against the PPN and at the same time would render no harm to EPN.

There are several studies on various products in which these were found to cause high juvenile mortality due to the presence of antimicrobial, nematostatic, or plant/animal additive compounds (Terefe et al., 2009; Miamoto et al., 2017). However, none of these studies could be considered complete, in the context of integrated pest management, as they included no reports regarding product effects on biological control agents and specifically EPN. Considering a broad range of biological control agents could be difficult, however studying the effects of these products on closely related to PPN beneficial organisms is more feasible and relevant. Therefore, the present study is unique as it explores effects of the product on both harmful and beneficial nematodes.

Under the conditions of this study, we conclude that the Alltech proprietary blend of fermentation and plant extracts with micronutrients was found to be an efficient, environmentally safe, and promising approach for PPN management.
